# Impact of Trocar Position on Surgical Site Infection After Pediatric Laparoscopic Appendectomy: A 15-Year Single-Center Study

**DOI:** 10.3390/medsci14020173

**Published:** 2026-03-31

**Authors:** Zenon Pogorelić, Mateo Kraljević, Ivan Lovrinčević, Ivan Maleš

**Affiliations:** 1 Department of Pediatric Surgery, University Hospital of Split, 21000 Split, Croatia; 2 Department of Surgery, School of Medicine, University of Split, 21000 Split, Croatia; 3 Department of Surgery, University Hospital of Split, 21000 Split, Croatia

**Keywords:** pediatric appendicitis, laparoscopic appendectomy, trocar placement, specimen extraction, surgical site infection, postoperative complications, specimen retrieval bag

## Abstract

**Background**: Surgical site infection (SSI) remains the most frequent postoperative complication after pediatric laparoscopic appendectomy. Evidence is scarce regarding whether the specimen extraction port site represents a modifiable risk factor. This study evaluated the association between 10 mm trocar placement for appendix extraction and postoperative outcomes in children undergoing laparoscopic appendectomy. **Methods**: A retrospective single-center cohort study was conducted including children aged 0–17 years who underwent laparoscopic appendectomy between January 2012 and January 2026 with ≥30-day follow-up. Patients were grouped by placement site of the 10 mm trocar: supraumbilical versus left lower quadrant (LLQ). The primary outcome was postoperative wound infection. Secondary outcomes included overall complications, intra-abdominal abscess, postoperative ileus, stump dehiscence, operative time, length of stay, readmission, reoperation, and conversion to laparotomy. Subgroup analyses assessed the impact of endoscopic retrieval-bag use within each trocar-position group. **Results**: Baseline demographic, clinical, laboratory, and histopathological characteristics were comparable between the two 10 mm trocar placement sites. Overall, postoperative complications were higher with supraumbilical placement than with LLQ placement (6.9% vs. 2.9%, *p* < 0.001). SSI was more frequent with supraumbilical placement (3.7% vs. 0.3%, *p* < 0.001). Multivariable analysis confirmed trocar position as an independent predictor of SSI, with LLQ placement associated with a lower risk (OR 0.52, 95% CI 0.30–0.88, *p* = 0.015). Operative time was shorter with LLQ placement (median 32 vs. 36 min, *p* < 0.001). No significant differences were observed between placement sites in intra-abdominal abscess, postoperative ileus, readmission, reoperation, conversion to laparotomy, or length of hospital stay. Retrieval-bag use was not associated with differences in complication rates within either trocar placement site. **Conclusions**: LLQ 10 mm trocar placement site was associated with substantially lower SSI rates and shorter operative time compared with supraumbilical extraction, without increasing other postoperative complications. Extraction port selection may represent a simple technical measure to improve outcomes in pediatric laparoscopic appendectomy without requiring additional resources.

## 1. Introduction

Acute appendicitis represents the most common surgical condition in children and is the leading cause of emergency abdominal operations in the pediatric population. It most frequently affects school-aged children and adolescents, and appendectomy remains the standard treatment for both uncomplicated and complicated presentations [1,2]. In the United States, it is estimated that approximately 60,000 to 80,000 pediatric appendectomies are performed each year [3]. Laparoscopic appendectomy is widely regarded as the preferred surgical technique for managing acute appendicitis in children, supported by numerous high-quality studies and clinical guidelines, and is associated with significantly faster postoperative recovery, shorter hospital stay, and reduced healthcare costs; moreover, the most recent studies indicate that children can be safely discharged on the same day after the surgical procedure [3,4]. The World Society of Emergency Surgery recommends the laparoscopic approach over open surgery in pediatric patients, highlighting benefits such as decreased postoperative pain, lower rates of surgical site infections (SSIs), shorter hospitalization, and better overall better quality of life when appropriate equipment and surgical expertise are available [5]. Large-scale registry data further demonstrate that laparoscopy is performed in more than 94% of pediatric appendectomies in the United States, with low rates of complications and conversion to open surgery [6].

Although laparoscopic appendectomy is considered the standard treatment for acute appendicitis in children, postoperative complications still occur. The most frequently reported complications include SSI, intra-abdominal abscess formation, bowel obstruction, hospital readmission, and the need for reoperation. Data from large population-based and registry studies indicate that the overall complication rate in pediatric patients ranges between 2% and 7%, with higher incidences observed in younger children and in cases of complicated appendicitis [7]. Surgical wound infection represents the most frequent and clinically significant complication after laparoscopic appendectomy in children, with reported incidence rates ranging from 1.3 to 4% in large pediatric studies and systematic reviews [8]. Despite laparoscopic appendectomy being established as the gold-standard technique, SSI remains the most common postoperative adverse event. Meta-analyses and analyses of national databases consistently demonstrate that the laparoscopic approach significantly reduces the risk of SSI compared with open appendectomy; however, it does not completely eliminate this complication [9]. The occurrence of SSI is linked to greater morbidity, longer hospitalization, the need for additional antibiotic therapy, and occasionally further surgical intervention [10].

Current clinical evidence indicates that complicated appendicitis is an independent risk factor for postoperative organ/space infection in children undergoing appendectomy, together with factors such as low preoperative lymphocyte-to-CRP ratio, pan-peritonitis, systemic inflammatory response syndrome, and abscess presentation. Additional factors associated with adverse postoperative outcomes after appendectomy include systemic inflammatory response, fever at presentation, drain use, and prolonged operative duration; however, these associations vary across studies and outcomes [11,12].

The World Society of Emergency Surgery advises the use of a single preoperative dose of prophylactic antibiotics in children with uncomplicated appendicitis, while recommending therapeutic antibiotic treatment for those with complicated disease. In such cases, antibiotics should also be initiated during the preoperative period if surgery cannot be performed immediately [5]. The use of endoscopic specimen-retrieval bags during laparoscopic appendectomy is linked to a reduced incidence of postoperative intra-abdominal abscess and is regarded as standard practice. By minimizing contamination during extraction of the appendix, these devices independently contribute to lower rates of intra-abdominal infections [13].

Insufficient research has been done in the literature on the effects of trocar placement on specimen extraction during laparoscopic appendectomy. High-quality research assessing the potential effects of extraction site selection on postoperative outcomes, such as SSI and other wound-related problems, is particularly lacking. In the pediatric population, where anatomical, immunological, and technical aspects may differ greatly from those in adults, this knowledge gap is particularly noticeable. As a result, the therapeutic implications of trocar position for children’s appendix extraction are still not well understood and need more thorough research. Therefore, optimizing the extraction port site may be an important technical component in lowering surgical wound problems following surgery. This study aims to investigate whether trocar placement is a separate modifiable risk factor impacting postoperative outcomes by examining the association between the extraction port’s location and the frequency of wound-related and intra-abdominal problems.

## 2. Methods

### 2.1. Patients

The medical records of 2474 pediatric patients who underwent appendectomy at the Department of Pediatric Surgery, University Hospital of Split, between 1 January 2012 and 1 January 2026 were retrospectively reviewed. Inclusion criteria were pediatric patients aged 0–17 years who underwent laparoscopic appendectomy for suspected acute appendicitis and had a minimum postoperative follow-up of 30 days. Patients who underwent open appendectomy, had incomplete medical documentation, had a follow-up period shorter than 30 days, had immunodeficiency, or in whom the operative report did not clearly describe trocar placement were excluded. After applying the exclusion criteria, a total of 1572 patients were eligible for further analysis.

Missing data were minimal and were handled using complete case analysis, with no formal imputation; analyses were conducted on available cases.

For the purpose of this study, patients were divided into two groups according to the location of the 10 mm trocar used for appendix extraction. Patients in whom the appendix was extracted through a supraumbilical 10 mm trocar were assigned to Group I (*n* = 712), while patients in whom the appendix was extracted through a left lower quadrant (LLQ) 10 mm trocar were assigned to Group II (*n* = 860).

**Figure 1:**
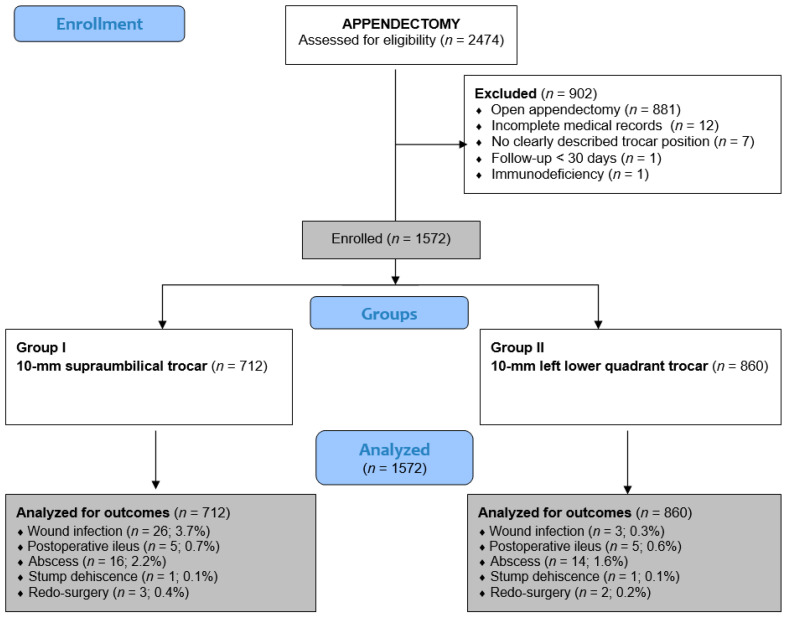
Flowchart of this study.

In additional analyses, each group was further subdivided according to whether an endoscopic retrieval bag was used for appendix extraction. The flowchart of patient selection is shown in Figure 1.

### 2.2. Ethical Aspects

This study was conducted in accordance with the Declaration of Helsinki and its later amendments. This study’s protocol was approved by the Institutional Review Board of the University Hospital of Split (approval number 520-03/26-01/14; date of approval 26 January 2026).

### 2.3. Outcomes of This Study

The primary outcome of this study was the incidence of surgical site infection (SSI), which was defined as any superficial or deep incisional infection diagnosed clinically during follow-up within 30 days postoperatively. Secondary outcomes included other postoperative complications (postoperative ileus, intra-abdominal abscess, stump dehiscence, bleeding, etc.), duration of surgery, length of hospital stay, readmission within 30 days, reoperation, and conversion to laparotomy. Postoperative complications were defined as events occurring within 30 days after the index operation.

### 2.4. Study Design

All patients underwent standard laparoscopic appendectomy for suspected acute appendicitis. The diagnosis was based on a combination of clinical examination, laboratory findings (white blood cell count, neutrophil percentage, and C-reactive protein), abdominal ultrasonography, and the Appendicitis Inflammatory Response (AIR) score [14]. The final decision to operate was made by the attending pediatric surgeon. Intraoperatively, appendicitis was classified as simple (catarrhal or phlegmonous) or complicated (gangrenous appendicitis, perforation, presence of pus in the abdominal cavity, or fecalith). The following data were collected for each patient: age, sex, height, body weight, body mass index, clinical presentation, laboratory parameters, operative details (trocar placement, duration of surgery, and use of an endoscopic retrieval bag), histopathological findings, postoperative complications, length of hospital stay, readmission, reoperation, and conversion to laparotomy.

In additional analyses, patients within each group were further stratified according to whether an endoscopic retrieval bag was used for appendix extraction.

### 2.5. Surgical Technique

**Figure 2:**
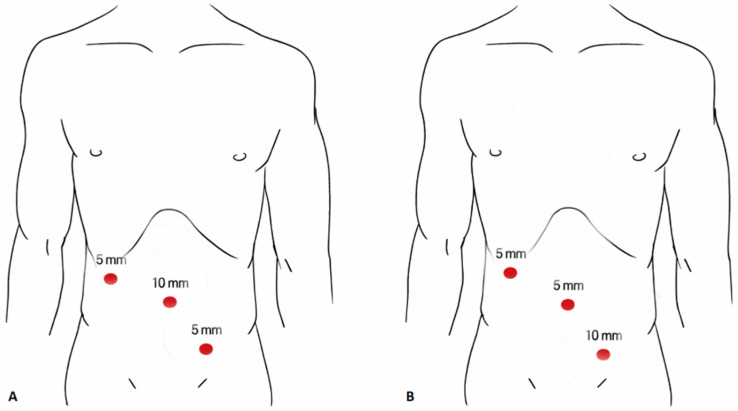
Schematic comparison of the two study groups: (**A**) Group I, 10 mm trocar above the umbilicus, 5 mm trocar below the right costal margin, and 5 mm trocar in the left lower quadrant. (**B**) Group II, 5 mm camera trocar above the umbilicus, 5 mm trocar below the right costal margin, and 10 mm trocar in the left lower quadrant used for appendix extraction without camera relocation.

All procedures were performed under general anesthesia with endotracheal intubation, with the patient placed in the supine position. Procedures were performed by a core team of 7 board-certified pediatric surgeons. Surgical residents were variably involved depending on rotation schedules, and all procedures involving residents were performed under direct supervision, reflecting routine clinical practice in a tertiary pediatric surgical center. Pneumoperitoneum was established using carbon dioxide at a pressure of 8–12 mmHg, depending on the patient’s age and body weight. In Group I (supraumbilical extraction group), a 10 mm trocar was placed in the supraumbilical position and used for appendix extraction. A 5 mm trocar was placed under the right costal margin in the midclavicular line, and an additional 5 mm trocar was placed in the LLQ. During appendix extraction, the laparoscopic camera was temporarily transferred from the supraumbilical trocar to the LLQ 5 mm trocar (Figure 2A). In Group II (LLQ extraction group), a 5 mm trocar was placed in the supraumbilical position for the laparoscopic camera, and a 5 mm trocar was placed under the right costal margin in the midclavicular line. The 10 mm trocar was positioned in the LLQ and used for appendix extraction, allowing extraction to be performed without relocation of the camera (Figure 2B). Laparoscopic appendectomy was performed using a standard technique described previously [15,16]. The use of an endoscopic retrieval bag for appendix extraction was not mandated by institutional guidelines and was left to the discretion of the operating surgeon, based on individual preference and intraoperative findings.

### 2.6. Postoperative Protocol and Follow-Up

Postoperatively, intravenous fluids were administered until oral intake was initiated. Oral feeding was usually started within a few hours after surgery. Postoperative analgesia consisted of paracetamol (15 mg/kg) and/or diclofenac (1 mg/kg) as required. Patients with simple appendicitis generally did not receive postoperative antibiotic therapy. In cases of complicated appendicitis, antibiotic therapy was administered according to institutional protocol and adjusted based on microbiological findings when necessary. Discharge criteria included absence of fever, adequate pain control, tolerance of oral intake, and no clinical signs of complications. All patients were followed up in the outpatient clinic at 7 and 30 days after discharge.

### 2.7. Statistical Analysis

Statistical analysis was performed using the Statistical Package for Social Sciences (SPSS), version 28.0 (IBM Corp., Armonk, NY, USA). The distribution of continuous variables was assessed using the Shapiro–Wilk test. Continuous variables were presented as mean ± standard deviation (SD) or median with interquartile range (IQR), as appropriate. Categorical variables were expressed as absolute numbers and percentages. Comparisons between groups were performed using the independent-samples *t*-test or Mann–Whitney U test for continuous variables, and the chi-square test or Fisher’s exact test for categorical variables, depending on expected cell counts. A two-sided *p*-value < 0.05 was considered statistically significant. To identify independent predictors of SSI, a multivariable logistic regression analysis was performed. Variables included in the model were trocar position (LLQ vs. supraumbilical), age, sex, presence of complicated appendicitis, and use of a specimen-retrieval bag. Results were reported as odds ratios (ORs) with 95% confidence intervals (CIs).

## 3. Results

A total of 1572 children who underwent laparoscopic appendectomy were included in this study. The median age of the entire cohort was 11 years (interquartile range (IQR), 9–15), and male patients predominated (approximately two thirds of the cohort). Median body weight and height were 45 kg (IQR, 34–61) and 158 cm (IQR, 142–174), respectively, with a median body mass index within the normal range. The median duration of symptoms prior to surgery was 24 h (IQR, 20–48). At presentation, most patients had localized right lower quadrant pain, frequently accompanied by rebound tenderness, while vomiting was present in more than half of the patients. The median Appendicitis Inflammatory Response (AIR) score was 7 (IQR, 4–8), indicating a moderate inflammatory response at admission. Laboratory evaluation revealed elevated inflammatory markers, with a median leukocyte count of 14.3 × 10^9^/L (IQR, 11.2–17.6), a median neutrophil percentage of 80.5% (IQR, 74.0–87.2), and a median C-reactive protein level of 17 mg/L (IQR, 6–45).

When patients were stratified according to the position of the 10 mm trocar, no significant differences were observed between the groups in demographic variables, clinical presentation, severity scores, or laboratory parameters (Table 1), indicating a well-balanced study population.

Histopathological diagnoses were similarly distributed between the groups (Table 2). Acute phlegmonous or suppurative appendicitis was the most common finding, followed by gangrenous appendicitis. The incidence of negative appendectomy, Enterobius vermicularis infestation, and neuroendocrine tumors was low and comparable between groups, with no statistically significant differences (*p* = 0.844).

**Figure 3:**
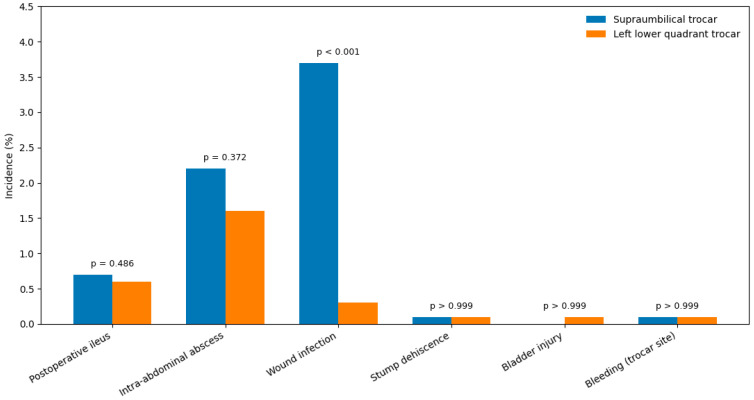
Postoperative complications according to trocar position.

Overall postoperative complications occurred in 49 patients (6.9%) in Group I and 25 patients (2.9%) in Group II, representing a significantly lower complication rate in the LLQ trocar group (*p* < 0.001). The rate of postoperative wound infection was significantly higher in Group I compared with Group II (3.7% vs. 0.3%, *p* < 0.001). No significant differences were observed between the groups regarding postoperative ileus, intra-abdominal abscess formation, stump dehiscence, bladder injury, or bleeding at the trocar insertion site (Figure 3).

The duration of surgery was significantly shorter in Group II compared with Group I (median 32 vs. 36 min, *p* < 0.001). Readmission, redo-surgery, and conversion to laparotomy rates were very low in both groups. In addition, the length of hospital stay did not differ significantly between the groups (Table 3).

Subgroup analysis according to the use of an endoscopic retrieval bag demonstrated no significant differences in overall complication rates or individual postoperative complications within either trocar-position group (Table 4). The use of a retrieval bag did not influence the incidence of wound infection, intra-abdominal abscess, stump dehiscence, or other postoperative complications.

Multivariable logistic regression analysis identified trocar position and complicated appendicitis as independent predictors of SSI. Extraction through a left lower quadrant trocar was associated with a significantly lower risk of SSI compared with supraumbilical extraction (OR 0.52, 95% CI 0.30–0.88, *p* = 0.015). In contrast, complicated appendicitis was associated with a markedly increased risk of SSI (OR 2.85, 95% CI 1.65–4.92, *p* < 0.001). Age, sex, and the use of a specimen-retrieval bag were not significantly associated with SSI in the adjusted model (Table 5).

According to the Clavien–Dindo classification, most postoperative complications in both groups were classified as Grade I or II. Severe complications (Grade IIIb) requiring reoperation occurred in five patients overall, with no significant difference between groups. No Grade IV or V complications were observed (Table 6).

## 4. Discussion

This study demonstrates that appendix extraction through a 10 mm left lower quadrant trocar during pediatric laparoscopic appendectomy is associated with a clinically significant reduction in SSI incidence and shorter duration of surgery compared with appendix extraction through a 10 mm supraumbilical trocar. This study’s groups were well balanced with key parameters affecting surgical outcomes, including demographic characteristics, clinical presentation, disease severity, laboratory findings, and histopathological diagnosis. This methodological balance strengthens the validity of the present results.

Importantly, multivariable analysis confirmed that trocar position is an independent predictor of SSI. Even after adjustment for age, sex, disease severity, and use of a specimen-retrieval bag, extraction through a left lower quadrant trocar remained associated with a significantly lower risk of SSI. Given that LLQ extraction requires no additional equipment and only minimal modification of standard port placement, it represents a practical approach that may improve wound-related outcomes in pediatric laparoscopic appendectomy. As expected, complicated appendicitis was independently associated with an increased risk of SSI, which is consistent with the previous literature.

Clinically, SSIs lead to prolonged hospital stay, higher readmission rates, and higher healthcare costs [17,18]. Adopting the LLQ as an extraction site is a simple technical modification that can be easily implemented in routine surgical practice without requiring additional resources and may improve patient outcomes. These findings support trocar position as a modifiable technical factor influencing postoperative outcomes.

The association of extraction through the LLQ trocar with a reduced incidence of SSI can be logically explained based on human anatomy and surgical technique. The primary mechanism underlying the reduced incidence of SSI is likely the avoidance of contamination from the umbilicus. Extraction of the appendix through a clean LLQ trocar, rather than through the bacteria-rich umbilicus, reduces the infectious inoculum at the wound site. This approach also minimizes tissue manipulation and skin contact. The shorter duration of surgery can be attributed to improved ergonomics and simplified workflow. The LLQ trocar allows a direct instrument path to the right iliac fossa, which facilitates appendiceal dissection and allows for a smoother and more efficient surgical workflow. The use of the LLQ trocar for extraction eliminates the need to move the camera during extraction, which prevents downtime during surgery. Together, these factors provide a reasonable explanation for both key findings.

Previous studies have shown that laparoscopic appendectomy is associated with a lower incidence of SSI compared with open appendectomy in pediatric and adult patients, including cases of complicated appendicitis [9,19,20]. The literature has shown that the incidence of SSI after laparoscopic appendectomy in pediatric patients is usually between 1% and 5%, depending on the severity of the disease, the antibiotic therapy used, and the definition of infection [21,22]. The overall incidence of SSI in our study is consistent with these data, serving as an additional indicator of the safety and external validity of our results. It also supports the overall safety profile of laparoscopic appendectomy in the pediatric population.

However, most previous studies focused on multiple strategies for SSI prevention, which included antibiotic management, extraction bags, or surgical technique. Very few publications have studied low-cost, widely available intraoperative interventions to reduce SSIs [23,24]. Therefore, our study meaningfully complements the current knowledge about SSI and enables the prevention of SSI with a low-cost method that can be widely applied.

The literature to date is extremely limited in its discussion of 10 mm trocar placement as an independent risk factor for SSI in children. Some studies in adult patients have analyzed trocar placement, but without a clear focus on SSI and without including the pediatric population [25]. This lack of data further emphasizes the importance of our study.

Previous studies have shown no significant difference in the incidence of SSIs between single-incision laparoscopic surgery and conventional laparoscopic appendectomy. This finding may be explained by the fact that, in most conventional laparoscopic appendectomy series, appendix extraction is routinely performed through the supraumbilical trocar [26,27,28]. This observation is consistent with the well-established higher infectious risk associated with umbilical incisions. Our results, therefore, do not contradict previous knowledge, but rather complement it.

In our study, the use of a specimen-retrieval bag during appendix extraction was not associated with a reduction in the incidence of SSI or other monitored postoperative complications. Turner et al. reported that the use of a retrieval bag during laparoscopic appendectomy was not associated with a statistically significant reduction in incidence of SSI, regardless of whether appendicitis was uncomplicated or complicated [24], which also fits the results of our study. In contrast, many authors have reported that the use of retrieval bags during laparoscopic appendectomy is associated with a significant reduction in SSIs and intra-abdominal abscess formation [13,29]. Overall, the existing literature reports inconsistent and often conflicting results regarding the impact of retrieval-bag use on postoperative outcomes, particularly in the pediatric population. While some large cohort studies have suggested a potential reduction in intra-abdominal infections with retrieval-bag use, others have demonstrated no significant difference in surgical site infection rates. These discrepancies may reflect heterogeneity in study design, patient populations, and the degree of intra-abdominal contamination. In this context, our findings suggest that extraction techniques, particularly the choice of extraction trocar, may play a more important role than the use of a retrieval bag alone in reducing postoperative complications [24,29,30]. Moreover, factors such as disease severity and intraoperative contamination appear to be stronger determinants of infectious outcomes than the extraction method itself [24,31,32].

Also, our study has some limitations that should be acknowledged. First, it is a retrospective single-center study, which may limit the generalizability of the findings to other institutions with different patient populations, surgical expertise, and institutional protocols. In addition, no randomization was used in determining the extraction site, and the choice of extraction port depended on individual surgeon preference, introducing a potential risk of selection bias. Although baseline characteristics were comparable between groups, residual confounding cannot be excluded, as unmeasured factors such as surgical technique, case complexity, perioperative management, and surgeon experience may have influenced both trocar selection and postoperative outcomes. Procedures were performed by multiple surgeons in an emergency setting, which may have introduced variability in surgical technique and perioperative decision-making. While this reflects real-world clinical practice, it may have contributed to heterogeneity in the observed outcomes. Furthermore, this study spans a 15-year period, during which surgical techniques and practice patterns may have evolved, and a temporal trend in trocar positioning cannot be excluded.

Despite these limitations, this study has significant strengths. The large sample size and extended follow-up period increase the reliability of the findings. Our results are consistent, clinically relevant, and supported by a clear anatomical explanation. It is also highly intuitive from a surgical perspective that avoiding extraction through the supraumbilical trocar could reduce the incidence of SSI.

The findings of this study have important implications for pediatric laparoscopic appendectomy. Given the observed reduction in SSI incidence and shorter operative time, extraction through a 10 mm LLQ trocar may represent a preferable technical option. Moreover, because this modification requires only minimal changes in trocar placement and does not increase costs, its implementation in routine practice would be straightforward.

From a broader perspective, these results imply that small, well-considered technical adjustments in minimally invasive surgery can significantly improve patient outcomes. Selection of extraction sites may represent an underappreciated but effective strategy for reducing postoperative complications. Future research should focus on prospective, multicenter studies to confirm these findings and improve their universality. In addition, further studies evaluating long-term outcomes and costs would provide valuable insight into the broader impact of this approach.

## 5. Conclusions

In this large retrospective cohort of children undergoing laparoscopic appendectomy, the location of the 10 mm trocar used for appendix extraction was associated with postoperative wound infection. Extraction through a left lower quadrant port was associated with a lower SSI rate compared with supraumbilical extraction and was additionally associated with a shorter operative time. Importantly, this technical modification was not associated with an increased risk of other clinically relevant adverse outcomes, including intra-abdominal abscess, postoperative ileus, stump dehiscence, readmission, reoperation, conversion to laparotomy, or length of hospital stay. Subgroup analyses suggested that the use of an endoscopic retrieval bag did not materially influence postoperative outcomes within either trocar-position strategy.

These findings suggest that extraction site selection is an underappreciated, modifiable intraoperative factor with meaningful clinical impact. Given that LLQ extraction requires no additional equipment and minimal adjustment of standard port placement, it represents a practical, cost-neutral strategy that may be considered to improve wound-related outcomes in pediatric laparoscopic appendectomy. Prospective, multicenter studies are warranted to confirm these results, evaluate potential confounding from surgeon preference and case complexity, and define optimal port strategies across uncomplicated and complicated appendicitis.

## Figures and Tables

**Table 1:** Baseline demographics, and clinical and laboratory characteristics of patients (*n* = 1572).

	Group I (*n* = 712)	Group II (*n* = 860)	*p*
	10 mm Supraumbilical Port	10 mm Left Lower Quadrant Port	
Demographic characteristics of patients			
Age (years)	12 (9, 15)	12 (9, 16)	0.632 *
Gender			0.611 †
Male	462 (64.9)	553 (64.3)	
Female	250 (35.1)	307 (35.7)	
Weight (kg)	46 (35, 62)	47 (36, 63)	0.719 *
Height (cm)	158 (142, 173)	160 (143, 174)	0.614 *
Clinical data of patients			
Duration of symptoms (h)	24 (18, 44)	26 (18, 50)	0.211 *
Body temperature (°C)	37.2 ± 0.7	37.2 ± 0.8	0.884 §
Vomiting	401 (56.3)	489 (56.9)	0.812 †
Pain in RLQ	712 (100)	860 (100)	>0.999 ‡
Rebound tenderness	583 (81.9)	717 (83.4)	0.113 †
AIR score	7 (4, 8)	7 (4, 8)	0.907 *
Laboratory data of patients			
Leukocytes (×10^9^/L)	14.6 ± 4.9	14.5 ± 5.1	0.768 §
CRP (mg/L)	17.2 (6.1, 42.3)	19.6 (6.4, 48.9)	0.181 *
Neutrophil granulocytes (%)	79.1 ± 10.4	82.4 ± 9.9	0.095 §

Data are presented as mean ± SD, median (IQR) or *n* (%) as appropriate. CRP—C-reactive protein; IQR—interquartile range; RLQ—right lower quadrant; AIR—Appendicitis Inflammatory Response; SD—standard deviation. *, Mann–Whitney U test; †, Chi-square test; ‡, Fisher’s exact test; §, independent *t*-test.

**Table 2:** Histopathological findings of appendicitis according to 10 mm trocar extraction site (*n* = 1572).

Histopathological Diagnosis, *n* (%)	Group I (*n* = 712)	Group II (*n* = 860)	*p*
	10 mm Supraumbilical Port	10 mm Left Lower Quadrant Port	
Acute appendicitis (phlegmonous/suppurative)	384 (53.9)	462 (53.7)	0.844 ^†^
Acute appendicitis (gangrenous)	258 (36.3)	318 (37.0)	
Chronic appendicitis	8 (1.1)	8 (0.9)	
Enterobius vermicularis	7 (1.0)	8 (0.9)	
Neuroendocrine tumor	3 (0.4)	4 (0.5)	
Negative appendectomy	52 (7.3)	60 (7.0)	

Data are presented as *n* (%), †, Global Fisher’s exact test.

**Table 3:** Treatment outcomes between groups according to 10 mm trocar placement (*n* = 1572).

Variables	Group I (*n* = 712)	Group II (*n* = 860)	*p*
	10 mm Supraumbilical Port	10 mm Left Lower Quadrant Port	
Complications (total)	49 (6.9)	25 (2.9)	<0.001 *
Postoperative ileus	5 (0.7)	5 (0.6)	0.486 †
Abscess	16 (2.2)	14 (1.6)	0.372 *
Wound infection	26 (3.7)	3 (0.3)	<0.001 †
Stump dehiscence	1 (0.1)	1 (0.1)	>0.999 †
Bladder injury	0 (0)	1 (0.1)	>0.999 †
Bleeding—trocar insertion site	1 (0.1)	1 (0.1)	>0.999 †
Duration of surgery (min)	36 (30–45)	32 (28–40)	<0.001 ‡
Readmission	12 (1.7)	10 (1.2)	0.508 *
Redo-surgery	3 (0.4)	2 (0.2)	0.664 †
Conversion to laparotomy	2 (0.3)	2 (0.2)	>0.999 †
Duration of hospital stay (days)	3 (2–5)	3 (2–4)	0.276 ‡

Data are presented as *n* (%) or median (IQR). *, Chi-square test; †, Fisher’s exact test; ‡, Mann–Whitney U test.

**Table 4:** Postoperative outcomes according to the use of an endoscopic retrieval bag, stratified by trocar position (*n* = 1572).

Variable	Group I—Supraumbilical 10 mm Trocar			Group II—Left Lower Quadrant 10 mm Trocar		
	Bag Used (*n* = 270)	No Bag (*n* = 442)	*p*	Bag Used (*n* = 328)	No Bag (*n* = 532)	*p*
Complications (total)	18 (6.7)	31 (7.0)	0.859 *	11 (3.4)	14 (2.6)	0.540 *
Wound infection	12 (4.4)	14 (3.2)	0.378 *	2 (0.6)	1 (0.2)	0.586 †
Intra-abdominal abscess	6 (2.2)	10 (2.3)	0.927 *	7 (2.1)	7 (1.3)	0.356 *
Stump dehiscence	0 (0.0)	1 (0.2)	>0.999 †	0 (0)	1 (0.2)	>0.999 †
Redo-surgery	2 (0.7)	1 (0.2)	0.566 †	1 (0.3)	1 (0.2)	>0.999 †
Conversion to laparotomy	1 (0.4)	1 (0.2)	>0.999 †	1 (0.3)	1 (0.2)	>0.999 †
Duration of surgery (min)	36 (30–45)	35 (29–44)	0.417 ‡	34 (28–41)	32 (27–40)	0.361 ‡

Data are presented as *n* (%) or median (IQR). *, Chi-square test; †, Fisher’s exact test; ‡, Mann–Whitney U test.

**Table 5:** Independent predictors of SSI after laparoscopic appendectomy.

Variable	OR	95% CI	*p*
Trocar position (LLQ vs. supraumbilical)	0.52	0.30–0.88	0.015
Age	0.98	0.92–1.04	0.52
Sex (male)	1.12	0.65–1.93	0.68
Complicated appendicitis	2.85	1.65–4.92	<0.001
Retrieval-bag use	0.76	0.44–1.31	0.32

LLQ—left lower quadrant; OR—odds ratio; CI—confidence interval.

**Table 6:** Clavien–Dindo classification of postoperative complications (*n* = 74).

Clavien–Dindo Grade	Group I (*n* = 49)	Group II (*n* = 25)	*p* *
	10 mm Supraumbilical Port	10 mm Left Lower Quadrant Port	
I	20 (40.8)	12 (48.0)	0.885
II	23 (46.9)	10 (40.0)	
IIIa	3 (6.1)	1 (4.0)	
IIIb	3 (6.1)	2 (8.0)	
IVa	0	0	
IVb	0	0	
V	0	0	

Data are presented as *n* (%). *, Global Fisher’s exact test.

## Data Availability

The original contributions presented in this study are included in the article. Further inquiries can be directed to the corresponding author.
